# Multiple maternal risk-management adaptations in the loggerhead sea turtle (*Caretta caretta*) mitigate clutch failure caused by catastrophic storms and predators

**DOI:** 10.1038/s41598-021-81968-0

**Published:** 2021-01-28

**Authors:** Deby L. Cassill

**Affiliations:** Department of Integrative Biology, USF, St. Petersburg campus, St. Petersburg, FL 33701 USA

**Keywords:** Behavioural ecology, Evolutionary ecology, Theoretical ecology, Ecology, Evolution, Zoology

## Abstract

Maternal risk-management, an extension of *r/K* selection, is an indispensable tool for understanding the natural selection pressures that shape the evolution of reproduction. Central to the construct of maternal risk-management is its definition of reproductive success as replacement fitness (*w* = 2), the survival of one breeding daughter to replace the female and one outbreeding son to replace her mate. Here, I apply maternal risk-management as a theoretical framework to explain multiple reproductive adaptations by loggerhead sea turtles nesting on a barrier island off the southern coast of Florida, US, from 1988 to 2004. Extrapolated over a 30-year reproductive span, nesting females averaged 4000–4500 eggs. I show that, rather than “putting all their eggs in one basket,” females divided eggs into 40 clutches of variable size (50–165 eggs). To deposit clutches, females migrated to the barrier island 10–12 times at unpredictable intervals of 2–8 years. Each nesting season, females deposited 1–7 clutches over diversified time intervals at diversified locations on the beach. Despite devastating clutch losses caused by ten catastrophic hurricanes, hundreds of erratic thunderstorms and dozens of predation events during this study, 72% of clutches produced by nesting females on this barrier island were undisturbed—median hatching success for these clutches was an astonishing 92%. I conclude that diversified maternal investments over time and space by nesting females are reproductive adaptations that have successfully offset clutch losses, thus enabling populations of loggerhead females to meet or exceed their reproductive goal of replacement fitness.

## Introduction

Reproduction is a fundamental process of biological systems. All organisms exist as a result of females repopulating ecosystems with the next generation of offspring. The majority of theories on the evolution of reproductive processes are optimality models with highly constrained assumptions that limit broad application^1–3^. In contrast, maternal risk-management, an empirical, bioeconomic model, reverses the top-down methodology of optimality models^4^. Central to the construct of maternal risk-management are three irrefutable facts: (1) in high-risk environments, breeding females overproduce offspring (i.e., Malthusian theory); (2) males diversify the offspring produced by females (i.e., sexual reproduction); (3) the vast majority of offspring perish before reaching sexual maturity (i.e., natural selection theory). According, to maternal risk-management, the reproductive goal of breeding females is replacement fitness (*w* = 2), the survival of at least one breeding daughter to replace her and at least one outbreeding son to replace her mate. Because replacement fitness is a constant, it applies universally to breeding females regardless of species, genus, family, order, class, or phylum. Depending on rates of mortality among their offspring, some breeding females fail to meet replacement fitness (*w* = 0). Other females meet or exceed replacement fitness (*w* ≥ 2). Across generations, populations evolve as changing environments selectively terminate the vast majority of offspring before they mature and reproduce.

**Figure 1:**
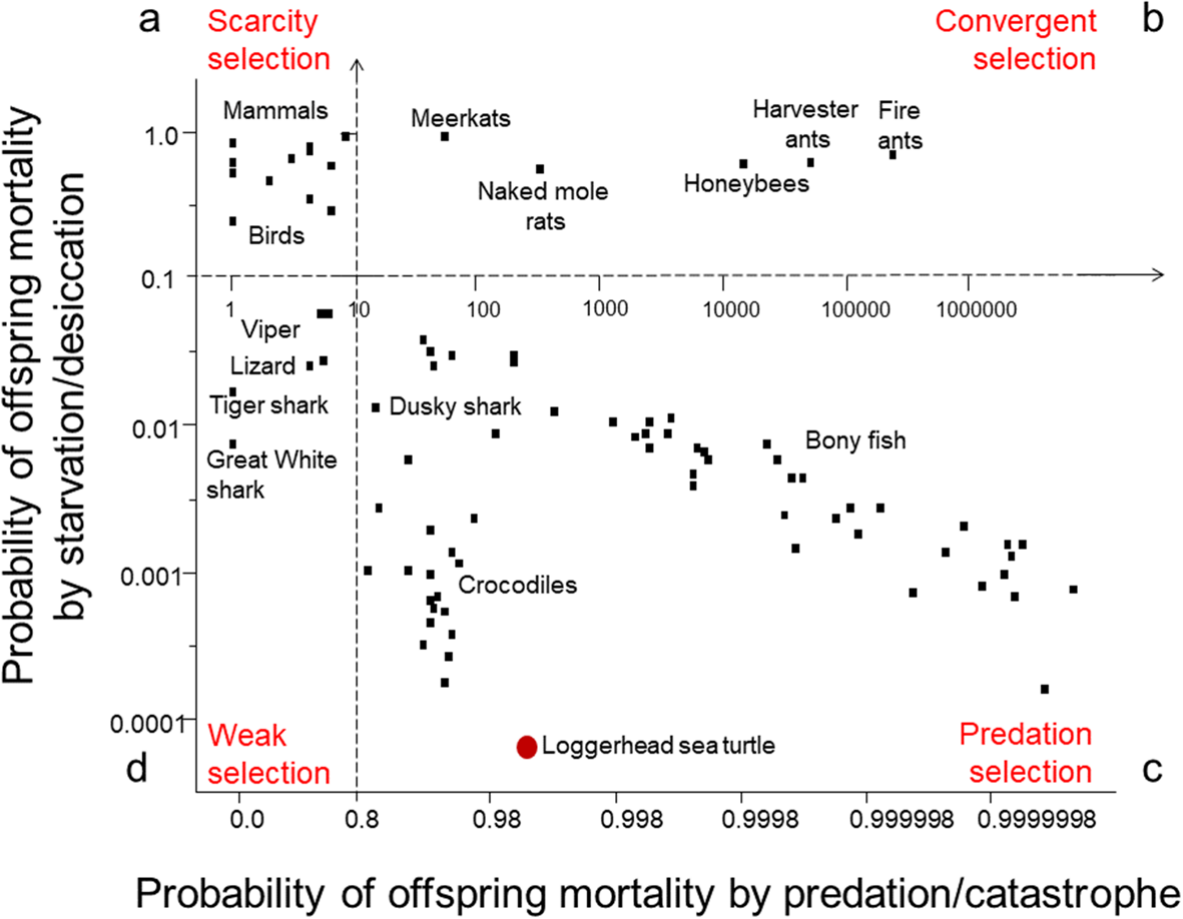
Maternal risk-management model (modified with permission^4^). The reproductive goal of breeding females is replacement fitness (*w* = 2). (**a**) Environments with a high probability of offspring starvation during seasonal cycles of scarcity select for females that invest in extended maternal care to a small number of offspring in temporary family units until offspring are capable of foraging or migrating on their own. Most birds and mammals are scarcity-selected species. (**b**) Environments with a high probability of offspring mortality by a combination of predation and starvation during seasonal cycles of scarcity select for the fusion of family units into hierarchical societies. During cycles of scarcity, those inside the margins provide resources to those at the margins to keep them alive and close at hand. During periods of high predation or invasions, those at the margins form a protective shield for those inside the margins. Additionally, those at the margins, the “canary in the coal mine,” are indicators of environmental toxins or infectious disease. Mammal societies and insect societies are convergent-selected species. (**c**) Environments with a high probability of offspring mortality by predation, disease or catastrophes select for females that overproduce, investing in large numbers of offspring. The loggerhead sea turtle is a predation–catastrophe-selected species. (**d**) Low-risk environments with low probabilities of predation or starvation select for females that invest in small number of precocial offspring that disperse at hatch or birth. The sand tiger shark is a weak-selected species.

Maternal risk-management generates a “map” displaying reproductive adaptations that mitigate offspring mortality by natural-selection processes (Fig. 1). With the maternal risk-management map as a unifying theoretical framework, we can explore the cost–benefit of divergent, species-specific adaptations that offset offspring mortality such as: capital breeders vs income breeders; oviparity versus viviparity; semelparity versus iteroparity; one-at-a-time versus thousands of offspring per breeding event; altricial versus precocial behavior of offspring at hatch or birth; equal versus skewed offspring size^5,6^; monogamy versus polygamy–polyandry–promiscuity; physical versus non-physical competitions for territories and mates; abandonment versus extended parental care; equal versus skewed sex ratio, and sexual reproduction versus parthenogenesis. Other reproductive adaptations include age of sexual maturity and reproductive longevity.

Among vertebrates, the dominant offspring-delivery system is oviparity, whereby females produce and expel amniotic eggs before embryonic development begins^7^. Eggs represent a significant investment by females to nourish the fertilized ovum through its growth and development from zygote to embryo to hatchling.

In oviparous birds and reptiles, the amniotic egg is a vessel containing the fertilized ovum, surrounded by layers of membrane-bound nutrients including yolk (composed of proteins, lipids, vitamins and minerals), albumen (composed of water and proteins), two membranes that defend against bacterial invasion, and a protective shell that is permeable to oxygen, carbon dioxide, and water vapor. After hatchlings emerge from the protective egg shell, extended maternal care occurs in nearly all bird species^8^ and is present among a number of reptile-groups^9–11^. However, extended maternal care of hatchlings is absent in one group of marine reptiles, the sea turtles.

Abandonment of clutches after eggs have been deposited is a highly-conserved reproductive adaptation in turtles, originating among the Testudines during the mid-Jurassic period, 174 million years ago^12–18^. Without extended care of eggs after they are deposited, females must deposit egg clutches on coastal beaches in semitropical-to-tropical regions with temperatures capable of supporting embryo growth and development inside the egg vessel^19–22^. Once hatchlings emerge from their subterranean nest and scuttle to the open sea, their survival depends on locating a suitable foraging area in tropical waters as well as finding refuge from predators^23,24^. Until recently, the probability of adult mortality was limited to disease^25,26^ and large sharks^27^. Today however, anthropogenic bycatch and pollution amplify the mortality of sea turtles at all stages of development from hatchlings to adults^28–32^.

Nesting females of the loggerhead sea turtle, *Caretta caretta*, are iteroparous breeders with a reproductive lifespan of 30 years^19^. At unpredictable intervals, females and males migrate to a mating area. The mating system is promiscuous and appears to be consensual during the period of time in which a female is sexually receptive to courtship by males^33–39^. After persistent interactions, males mount receptive females by clasping the edges of a female’s carapace with a single large claw on each flipper. Once attached to the female’s carapace, the male curves his tail under the female’s carapace and probes with his penis until he achieves penetration of the female’s cloaca^40^. Ovulation occurs only after the successful transmission of sperm by one or more males^41^. After the mating season is over, females migrate to their natal beach area; males return to foraging areas^19,42–46^.

For a large animal with long, tapered limbs designed for long-distance navigation across seas and oceans, nesting on a coastal beach is an arduous task for loggerhead females^47^. Upon emerging from the sea, each female crawls up the beach toward the higher-sloped, line of vegetation. Once a nest-site is selected, she excavates a large body pit using her fore-flippers. Then, using only her back flippers, she excavates a smaller pit into which dozens of eggs are expelled. Thereafter, females refill the pit with sand, re-shape and smooth the surface, and then camouflage the body pit by flipping layers of sand and debris until the surface is less noticeable^47^. In its entirety, nesting takes 30–120 min before the female abandons the clutch and returns to the sea^19^. During each nesting season, females deposit multiple clutches at intervals of 2–3 weeks over a 2–3 month nesting period^48–50^. Once their reproductive task is completed, females abandon their natal beach area and migrate back to foraging areas.

Along the coasts of Florida, US, the spring-to-fall nesting and hatching season of the loggerhead sea turtle overlaps with the spring-to-fall thunderstorm and hurricane season^51^. Torrential thunderstorms and tidal surges suffocate eggs in flooded sediment^52^. Strong currents and eroding beaches caused by catastrophic hurricanes and severe storms wash clutches out to sea^53^. In addition, storms expose clutches to desiccation^54^ and predators, including raccoons, domestic dogs, coyotes, sea birds, fish, ghost crabs and fire ants^55–60^. How do nesting loggerhead females offset the loss of clutches to severe storms and predators after clutches are abandoned?

Maternal risk-management classifies the loggerhead sea turtle as is a predation-catastrophe-selected species that must overproduce offspring to offset high rates of mortality (see Fig. 1). Because the relative size of offspring at dispersal is a small fraction of female size (egg size/female size), the model predicts that the probability of starvation is not a major source of offspring mortality. In contrast, because the number of eggs per clutch or per lifetime is relatively large, the model predicts that the probability of offspring mortality by predation is moderately high (1—*w*/number of eggs per clutch or per lifetime). Predation and storms are the primary sources of offspring mortality for eggs and hatchlings on the beach. Once hatchlings enter the oceanic environment, the risk of predation is again high.

What are the reproductive adaptations that offset the loss of egg clutches to storms and predator for the loggerhead sea turtle? Here, I present a long-term study, 1988–2004, on the maternal investments by loggerhead females nesting on a barrier island off the southern coast of Florida, US. The identities of the population of 112 loggerhead females and the fate of their 690 clutches were monitored and recorded every nesting season for 17 years. Hence, this study presents a rare opportunity to characterize the reproductive behaviors by individual females and the fate of their clutches over multiple, consecutive nesting seasons.

The first objective of this study was to characterize the patterns of storms on the barrier island and the impact of storms and predators on the hatching success of clutches deposited on the island over 17 nesting seasons. The second objective was to characterize the maternal investments by loggerhead females over 17 nesting seasons. The third objective was to determine which of a female’s investment strategies was most successful in mitigating clutch failure caused by storms and predators after clutches were deposited and abandoned.

## Results

In this first section, I report the frequency of hurricanes during the 17-year study and the impact of hurricanes and predators on clutch-hatching success.

### Pattern and impact of hurricanes on clutch-hatching success, 1988–2004

**Figure 2:**
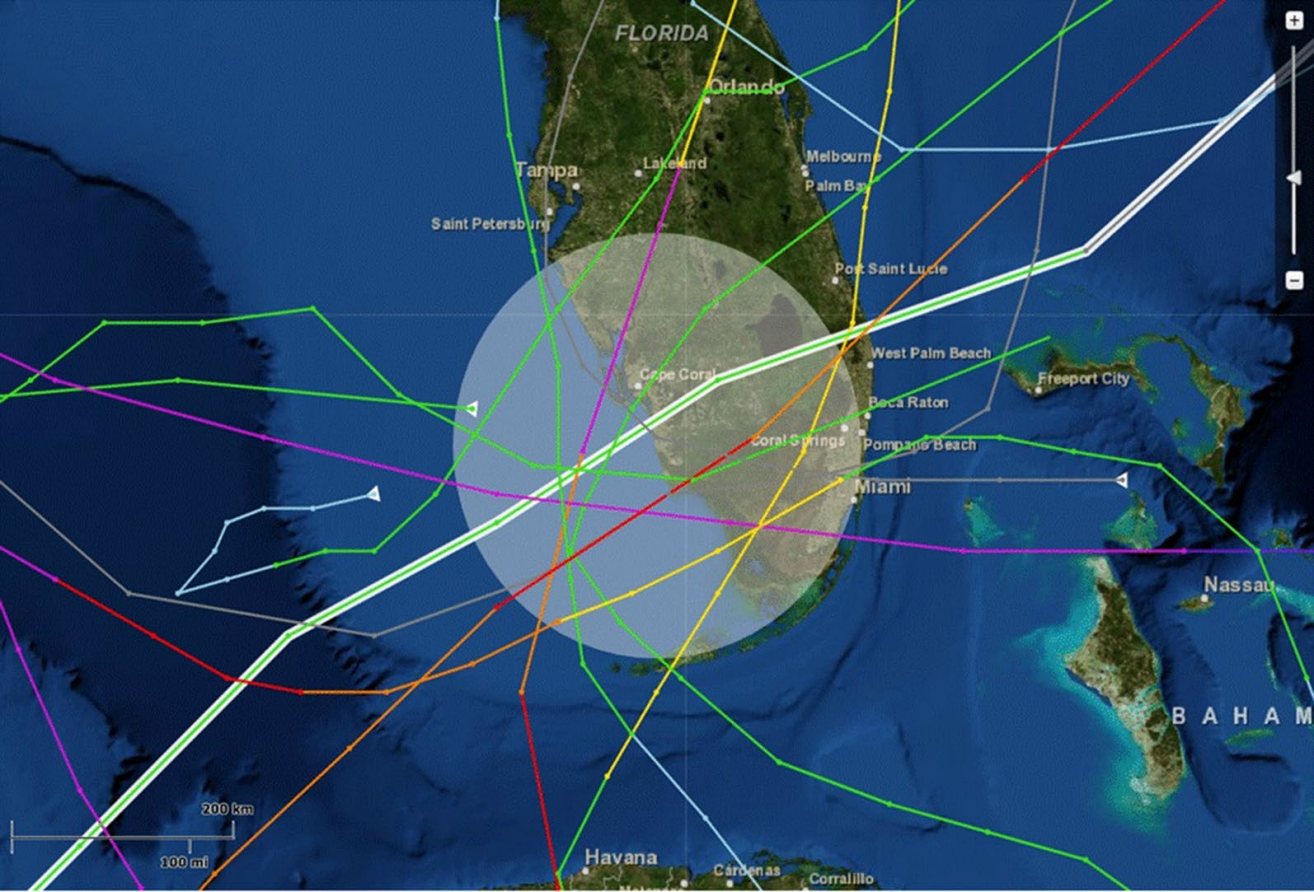
Path of hurricanes affecting the Gulf coast of south Florida, US, 1992–2004. This map was configured using NOAA’s Historical Hurricane Tracks, a free online tool developed by the NOAA Office for Coastal Management in partnership with NOAA’s National Hurricane Center and National centers for Environmental Information https://oceanservice.noaa.gov/news/historical-hurricanes/.

Ten hurricanes swept over the Gulf coast of south Florida, affecting the barrier island beaches during six of this 17-year study. Hurricane Andrew struck southern Florida in August 1992^61^; Hurricane Erin and Opal struck in August and October 1995; Hurricane Georges struck in September 1998; Hurricane Irene struck in October 1999; Hurricane Gabrielle struck in September 2001; and Hurricanes Charley, Frances, Ivan, and Jeanne struck in August and September, 2004 (Fig. 2).

**Figure 3:**
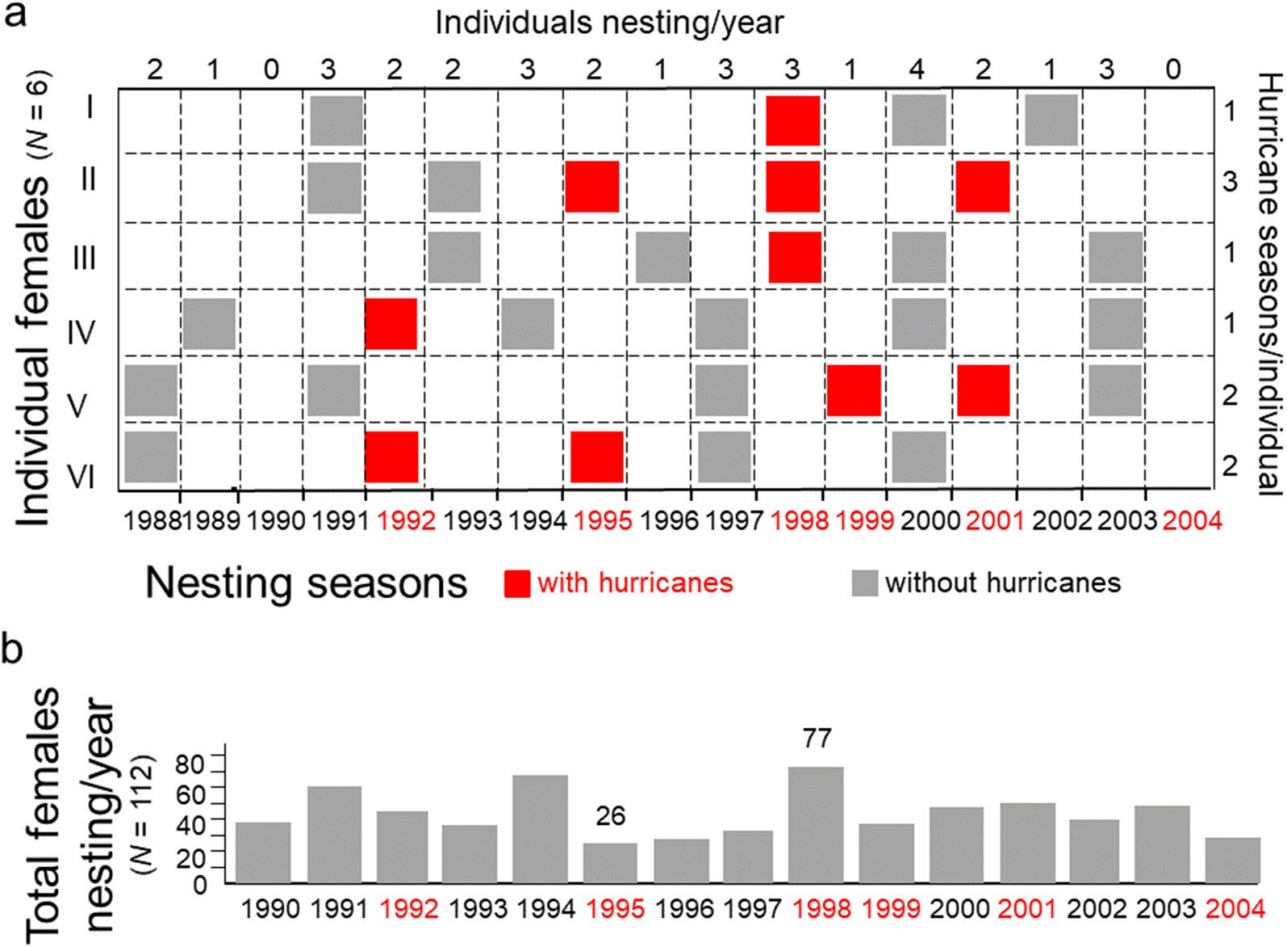
Remigration intervals and nesting seasons with hurricanes, 1988–2004. (**a**) Patterns of nesting seasons on the barrier island per individual (*N* = 6 females). (**b**) Number of females nesting each year on the barrier island (*N* = 112 females).

To assess the probability that individual females would nest during a season with hurricanes, I report the frequency of nesting seasons and the number of years between nesting seasons (i.e., remigration intervals) by females that migrated to the barrier island at least four times during the 17-year study (*N* = 6). For these individuals, remigration intervals ranged from 2 to 7 years with a median of 3 years (Fig. 3a). The number of times a female nested on the barrier island during a season with hurricanes ranged from 1 to 3. The number of times a female nested during a season without hurricanes ranged from 2 to 5. Remigration intervals per female were unpredictable, ranging from 2 to 8 years with a median of 3 years. As a result, the total number of females nesting each year on the barrier island was unpredictable, varying from 26 to 77 with a mean of 45 females per year (Fig. 3b).

**Figure 4:**
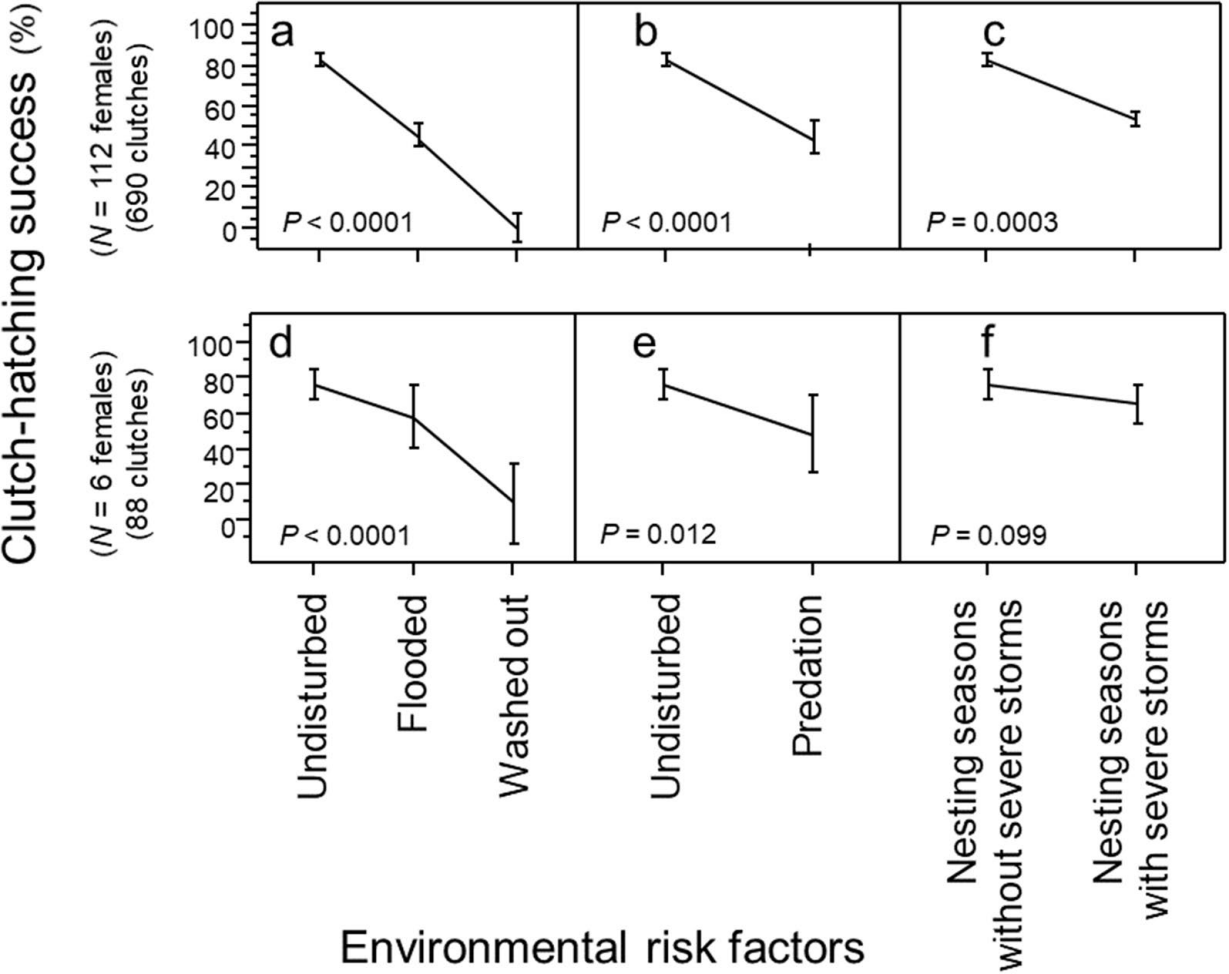
Clutch-hatching success relative to environmental risk factors, 1988–2004. (**a**) Clutch-hatching success by storms (*N* = 112 females). (**b**) Clutch-hatching success by predation (*N* = 112 females). (**c**) Clutch-hatching success by years with hurricanes (*N* = 112 females). (**d**) Clutch-hatching success by storms (*N* = 6 females). (**e**) Clutch-hatching success by predation (*N* = 6 females). (**f**) Clutch-hatching success by years with hurricanes (*N* = 6 females).

To what extent did storms and predators impact clutch-hatching success? For the population of females, predators, flooding, and washout significantly reduced clutch-hatching success (Fig. 4a–c; Multi-factor Chi-Square Approximation: *χ*^2^_4,607_ = 139.45; *R*^2^ = 0.45; *p* < 0.0001; *N* = 112 females). Storms accounted for 89.3% of explained variation in low clutch-hatching success; predation accounted for 10.7%. For the individual females, the patterns of hatching success or failure were comparable to that of the population of females (Fig. 4d–f; *N* = 6 females).

How do nesting females mitigate clutch failure? In the second section, I report patterns in maternal investments, spatial investments and temporal investments by loggerhead females at the population and individual levels.

### Patterns of maternal, spatial and temporal investments by nesting females, 1988–2004

For ease of reference, reproductive investments by loggerhead females were grouped into three categories (Table 1): (1) Maternal investments included mate number, clutch size (number of eggs deposited per clutch), number of clutches produced per nesting season, and fertility (the sum of eggs deposited among multiple clutches per female per nesting season). (2) Spatial investments included migration routes per nesting season, clutch location on the beach (nest-site selection per clutch), clutch distance from the vegetative line, clutch distance from the high-tide line, the depth of the beach, distance along the length of the beach from a permanent marker, distance between clutches along the beach, and nest depth. (3) Temporal investments included nesting month, clutch intervals (number of days between clutch oviposition within the same nesting season), and remigration intervals.

**Table 1:** Maternal-investments, spatial investments, and temporal investments by nesting females in the loggerhead sea turtle, *C. caretta*, 1988–2004. As noted, data on mate number, migration routes and nest depth were from other studies.

		Range	Mean
**Maternal investment adaptations**			
1	Mate number^34,39,62,63^	1–7 males	2.6
2	Clutch size	47–165	105
3	Number of clutches per nesting season	1–7	3
4	Fertility per nesting season (total egg number per female per nesting season)	193–733	289
**Spatial investment adaptations**			
5	Migration routes to mating areas^35,37^	Variable	
6	Clutch distance from the vegetative line (m)	− 8.2–22.9	1.2
7	Clutch distance from the high tide line (m)	0.0–41.8	17.0 m
8	Clutch deposition by depth of beach (m)	6.1–43.0	17.0 m
9	Clutch deposition along the beach (km)	1.5–12.2	9.0 m
10	Distance between clutches along the beach (m)	258–1953	732 m
11	Nest depth (cm)^64–66^	35–55	NA
**Temporal investment adaptations**			
12	Nesting season (month)	April–August	June
13	Clutch intervals per nesting season (number of days)	10–32	15
14	Remigration intervals (number of years)	2–8	3

**Figure 5:**
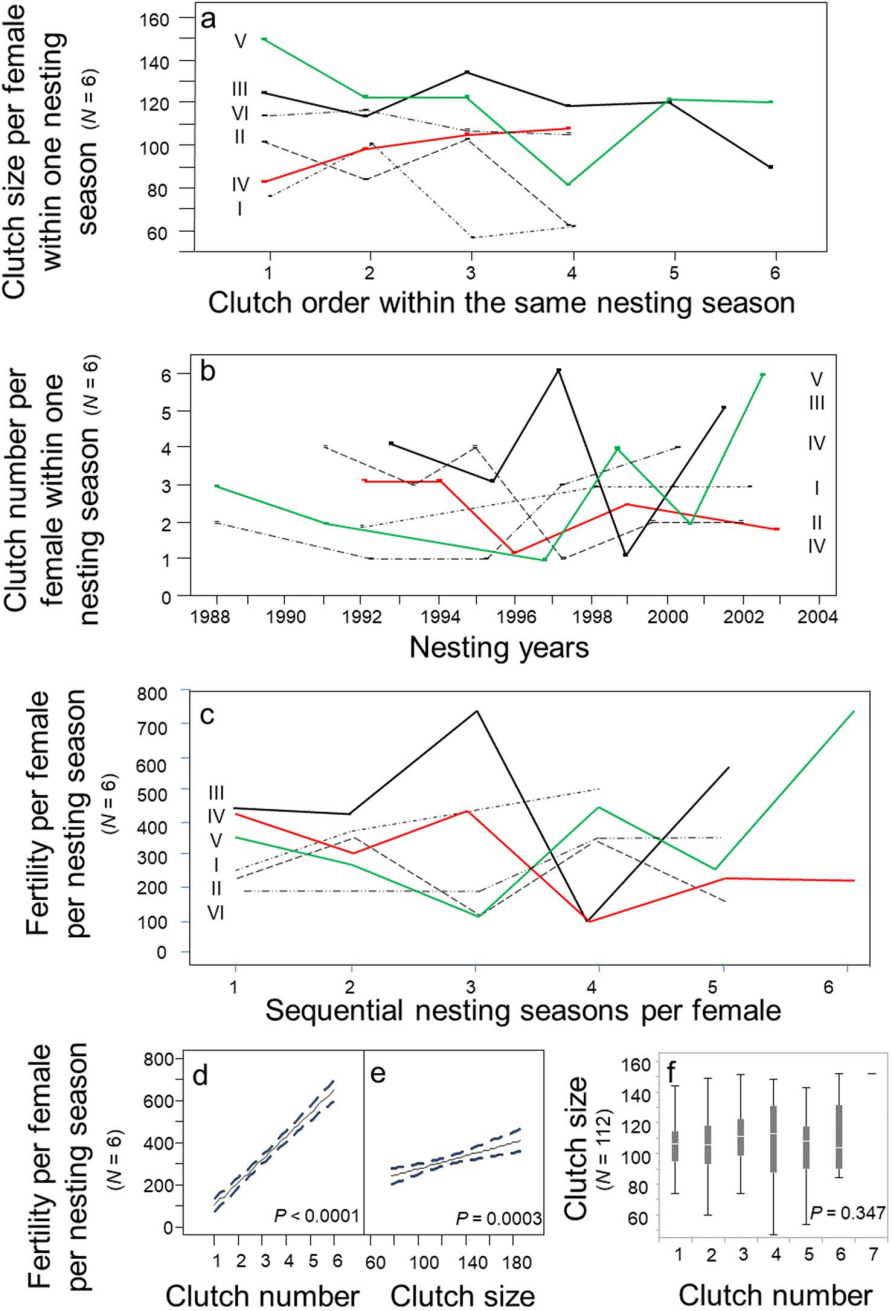
Temporal investments in fertility, 1988–2004. (**a**) Clutch size per female within a single nesting season (*N* = 6 females). (**b**). The number of clutches deposited per female within a single nesting season (*N* = 6 females). (**c**) Fertility per female per nesting season (*N* = 6 females). (**d**) Fertility by the number of clutches per female per nesting season (*N* = 6 females). (**e**) Fertility by clutch size per female per nesting season (*N* = 6 females). (**f**) Clutch size by number of clutches per nesting season (*N* = 112 females).

For a detailed analysis of fertility patterns, six females were selected from the population for which long-term maternal investments were available. Clutch size per female varied unpredictably within a single nesting season (Fig. 5a). The number of clutches produced per female per nesting season varied unpredictably over multiple nesting seasons (Fig. 5b). Female fertility within a nesting season varied unpredictably over multiple nesting seasons (Fig. 5c). The number of clutches was a stronger predictor of fertility per nesting season (Fig. 5d; Regression: *R*^2^ = 0.87; *p* < 0.0001; *N* = 112 females) than clutch size (Fig. 5e; Regression: *R*^2^ = 0.14; *p* = 0.042; *N* = 112 females). The number of clutches per nesting season accounted for 87% of explained variation in fertility per nesting season; clutch size accounted for the remainder. Lastly, clutch size and the number of clutches per nesting season were independent maternal investment strategies (Fig. 5f; ANOVA: *F*_6,268_ = 1.12; *p* = 0.347; *N* = 112 females).

I extrapolated information from the six aforementioned females and estimated that, over a 30-year reproductive lifespan, loggerhead females would produce an average of 4223 eggs divided into an average of 40 clutches. Nesting females would migrate to the barrier island to nest a total of 12 times with an interval averaging 3 years between migrations. Each nesting season, females would produce an average of three-to-four clutches over 2–3 months with an average of 105 eggs per clutch.

**Figure 6:**
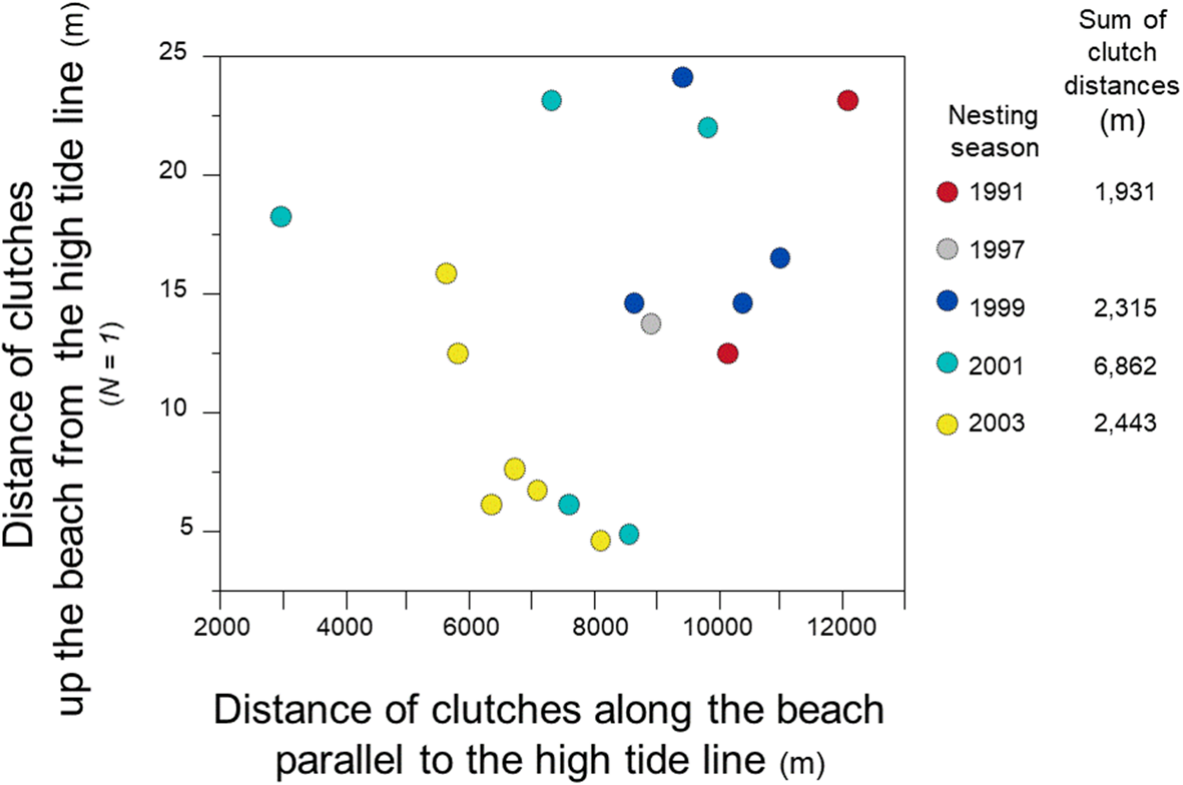
Nest-site selection, 1991–2003, *N* = 1 female. Distance of clutches from each other, deposited by one female within and among five nesting seasons. Data for clutch locations in 1988 were not available. In 1991, only one clutch for this female was observed and recorded.

When searching for nest sites on the beach, females emerge from the water and crawl up the beach toward the vegetative line, perpendicular to the high-tide line. For a detailed analysis of clutch locations within and among nesting seasons, I selected one female with a record of six nesting seasons from 1988 to 2003. Rather than clustering clutches on the beach, this female dispersed the location of clutches within and across nesting seasons (Fig. 6).

**Figure 7:**
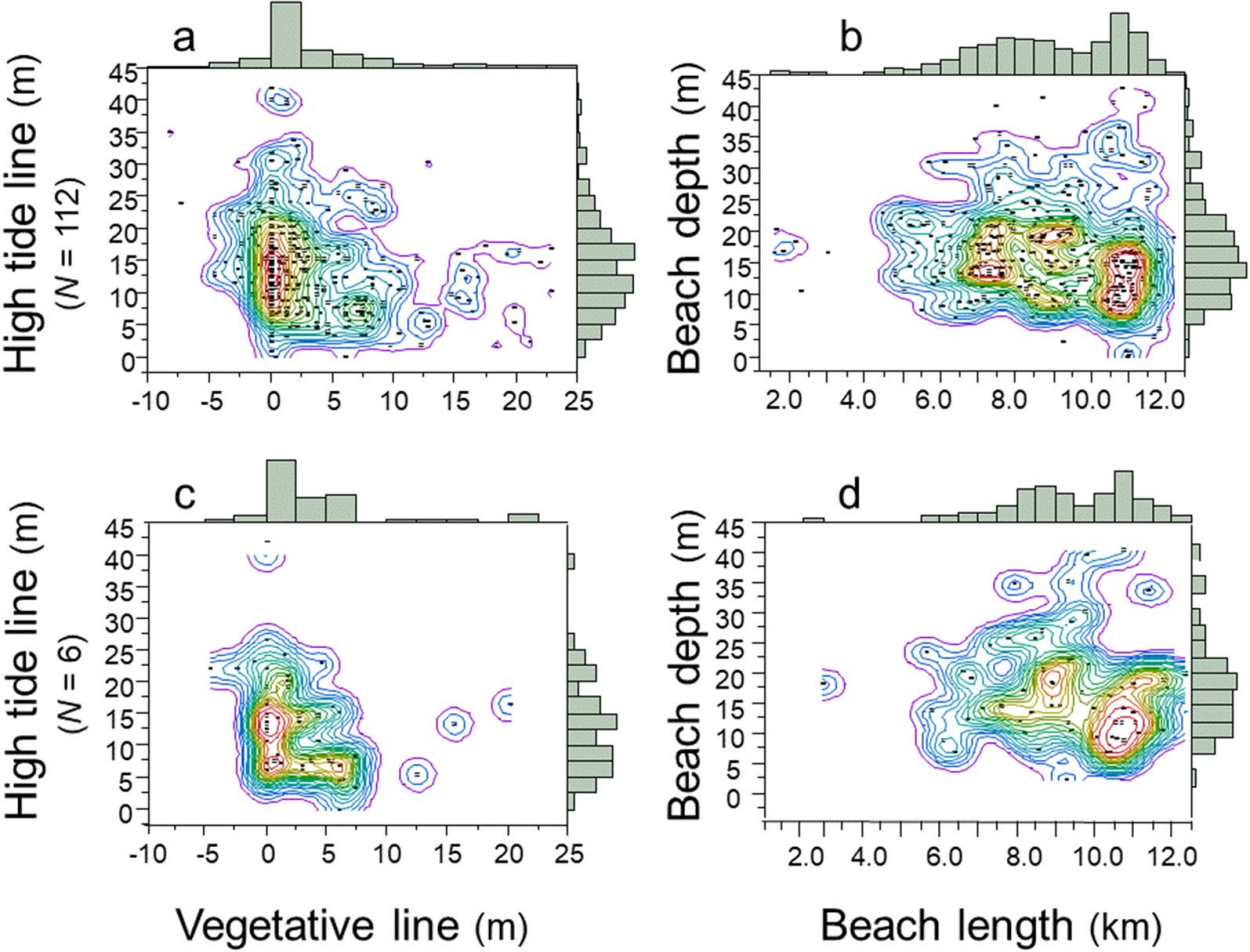
Density of nest-site selection, 1988–2004. (**a**) Distribution of clutches inside and outside the vegetative and high-tide Goldilocks zones (*N* = 112 females). (**b**) Distribution of clutches inside and outside the beach-depth-and-length Goldilocks zones (*N* = 112 females). (**c**) Distribution of clutches inside and outside the vegetative and high-tide Goldilocks zones (*N* = 6 females). (**d**) Distribution of clutches inside and outside the beach-depth-and-length Goldilocks zones (*N* = 6 females). These are JMP Pro15 density contour maps; the warm-colored contour lines delineate the Goldilocks zones.

Density contour maps show the diversified location of clutches relative to their distance from the vegetative line, the high-tide line, beach depth, and the length of the beach. I identified four regions that I refer to as “Goldilocks zones” within which the majority of clutches were deposited by the population of loggerhead females on this barrier island. The high-tide Goldilocks zone was 5–20 m of the high-tide line, within which 73.5% of clutches were deposited; the vegetative Goldilocks zone was a narrow ± 5 m of the vegetative line, within which 64.7% of clutches were deposited (Fig. 7a; *N* = 112 females). The beach-depth Goldilocks zone was a broad area of 5–25 m, within which 84.3% of clutches were deposited; the beach-length Goldilocks zone was a lengthy area from 6–13 km, within which 94.0% of clutches were deposited (Fig. 7b; *N* = 112 females). The Goldilocks zones for individual females were similar in pattern to those for the population of females (Fig. 7c,d; *N* = 6 females). Thus, the dispersed spatial distribution of clutches by females at the population level were emergent properties of the dispersed spatial distribution of clutches by individual females.

**Figure 8:**
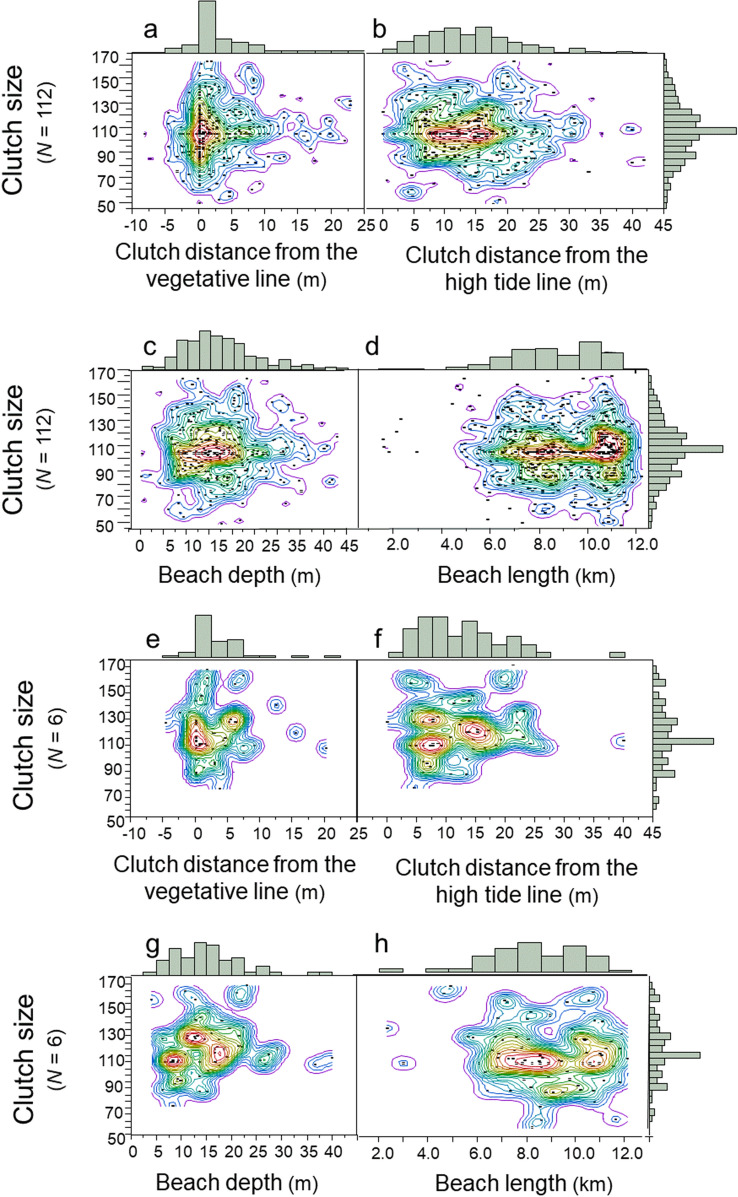
Clutch size by nest-site selection, 1988–2004. (**a**) Distribution of clutch size inside and outside the vegetative Goldilocks zone (*N* = 112 females). (**b**) Distribution of clutch size inside and outside the high-tide Goldilocks zone (*N* = 112 females). (**c**) Distribution of clutch size inside and outside the beach-depth Goldilocks zone (*N* = 112 females). (**d**) Distribution of clutch size inside and outside the beach-length Goldilocks zone (*N* = 112 females). (**e**) Distribution of clutch size inside and outside the vegetative Goldilocks zone (*N* = 6 females). (**f**) Distribution of clutch size inside and outside the high-tide Goldilocks zone (*N* = 6 females). (**g**) Distribution of clutch size inside and outside the beach-depth Goldilocks zone (*N* = 6 females). (**h**) Distribution of clutch size inside and outside the beach-length Goldilocks zone (*N* = 6 females). These are JMP Pro 15 density contour maps.

Do females deposit larger clutches inside the Goldilocks zones and smaller clutches outside the Goldilocks zones? For the population of females, clutch size was independent of clutch location relative to the vegetative line, the high-tide line, beach depth and beach length (Fig. 8a–d; Multivariate model: *F*_3,384_ = 2.44; *R*^2^ = 0.02; *p* = 0.064; *N* = 112 females). Clutch size and clutch location by individuals were similar in pattern to that of the population (Fig. 8e–h; *N* = 6 females, 88 clutches). In total, the diversified patterns of clutch size and nest-site selection at the population level were emergent properties of the diversified patterns of clutch size and nest-site selection at the individual level.

In the third and final section, I report whether or not females modified maternal, spatial, or temporal investments during nesting seasons with hurricanes. In addition, I report the impact of maternal, spatial, and temporal investments on clutch-hatching success. Lastly, I compare the impact of nesting seasons with hurricanes on the number of clutches that were disturbed by predation, flooding, or washout.

### Impact of maternal, temporal, and spatial investments on clutch hatching success, 1988–2004

Nesting females did not modify clutch size or the number of clutches during nesting seasons with hurricanes (t-test: t-ratio = 0.07; *p* = 0.946; Pearson: χ^2^ = 2.27; *p* = 0.893; *N* = 690). Nesting females did not modify the month of clutch production during nesting seasons with hurricanes (Pearson: χ^2^ = 2.30; *p* = 0.317). Nesting females did not modify clutch location by depth of beach during nesting seasons with hurricanes (t-test: t-ratio = 1.54; *p* = 0.122). Nesting females did not modify the distance of clutches from the vegetative line, or the distance of clutches from the high-tide line during nesting seasons with hurricanes (t-test: t-ratio = 0.07; *p* = 0.946; t-ratio = 1.36; *p* = 0.172). Lastly, the number of nesting females on the barrier island did not differ during nesting seasons with hurricanes (Chi Square Approximation: *χ*^2^ = 0.54; *p* = 0.462). In total, nesting females did not modify maternal, temporal, or spatial investments in anticipation of hurricanes.

Maternal investments in fertility were not a significant predictor of clutch-hatching success (Regression: R^2^ = 0.005; *p* = 0.715; *N* = 6 females). For example, a highly-fertile female (*N* = 5568 egg per lifetime) experienced low clutch-hatching success (53.1%), whereas a low-fertility female (*N* = 2822 eggs per lifetime) experienced high clutch-hatching success (87.1%). The number of surviving hatchlings for these two females was 2957 and 2458 respectfully.

**Figure 9:**
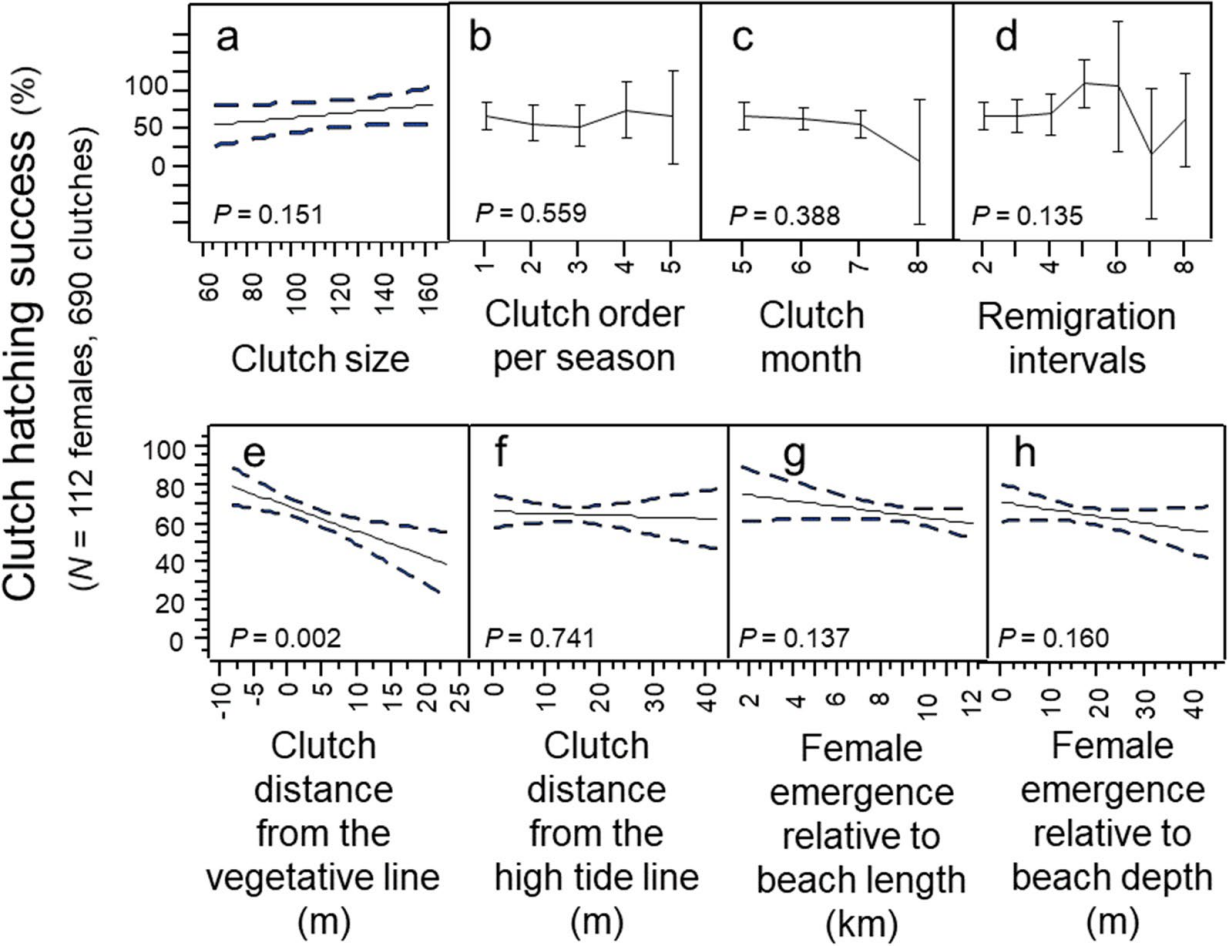
Clutch-hatching success by maternal, temporal, and spatial investment patterns, 1988–2004, *N* = 112 females. (**a**) Clutch-hatching success by clutch size. (**b**) Clutch-hatching success by the order in which clutches were deposited. (**c**) Clutch-hatching success by the month in which clutches were deposited. (**d**) Clutch-hatching success by remigration intervals. (**e**) Clutch-hatching success by clutch distance from the vegetative line. (**f**) Clutch-hatching success by clutch distance from the high-tide line. (**g**) Clutch-hatching success by the location of a female’s emergence relative to beach length. (**h**) Clutch-hatching success by the location of a female’s emergence relative to beach depth.

Clutch-hatching success did not differ significantly by clutch size, clutch order, clutch month, or remigration intervals (Fig. 9a–d; Multi-factor Chi-Square Approximation: *χ*^*2*^_13,126_ = 5.24; *R*^2^ = 0.11; *p* = 0.256; *N* = 112 females). Hatching success did not differ significantly based on clutch distance from the high-tide line, beach depth or beach length; and although hatching success differed significantly based on clutch distance from the vegetative line this variable accounted for only 2.6% of explained variation in hatching success (Fig. 9e–h; Multivariate model: *F*_3,377_ = 4.05; *R*^2^ = 0.03; *p* = 0.007; *N* = 112 females).

To further investigate clutch-hatching success relative to the vegetative Goldilocks zone, I classified the bimodal distribution of clutch-hatching success into two categories: high-hatch clutches (80–100% of eggs hatched); and failed clutches (0–20% of eggs hatched). I found that 63% of clutches inside the vegetative Goldilocks zone experienced high-hatch compared to 50% of clutches outside the zone. In addition, just over 16% of clutches inside the vegetative Goldilocks zone experienced clutch failure compared to 33% of clutches outside the zone.

**Figure 10:**
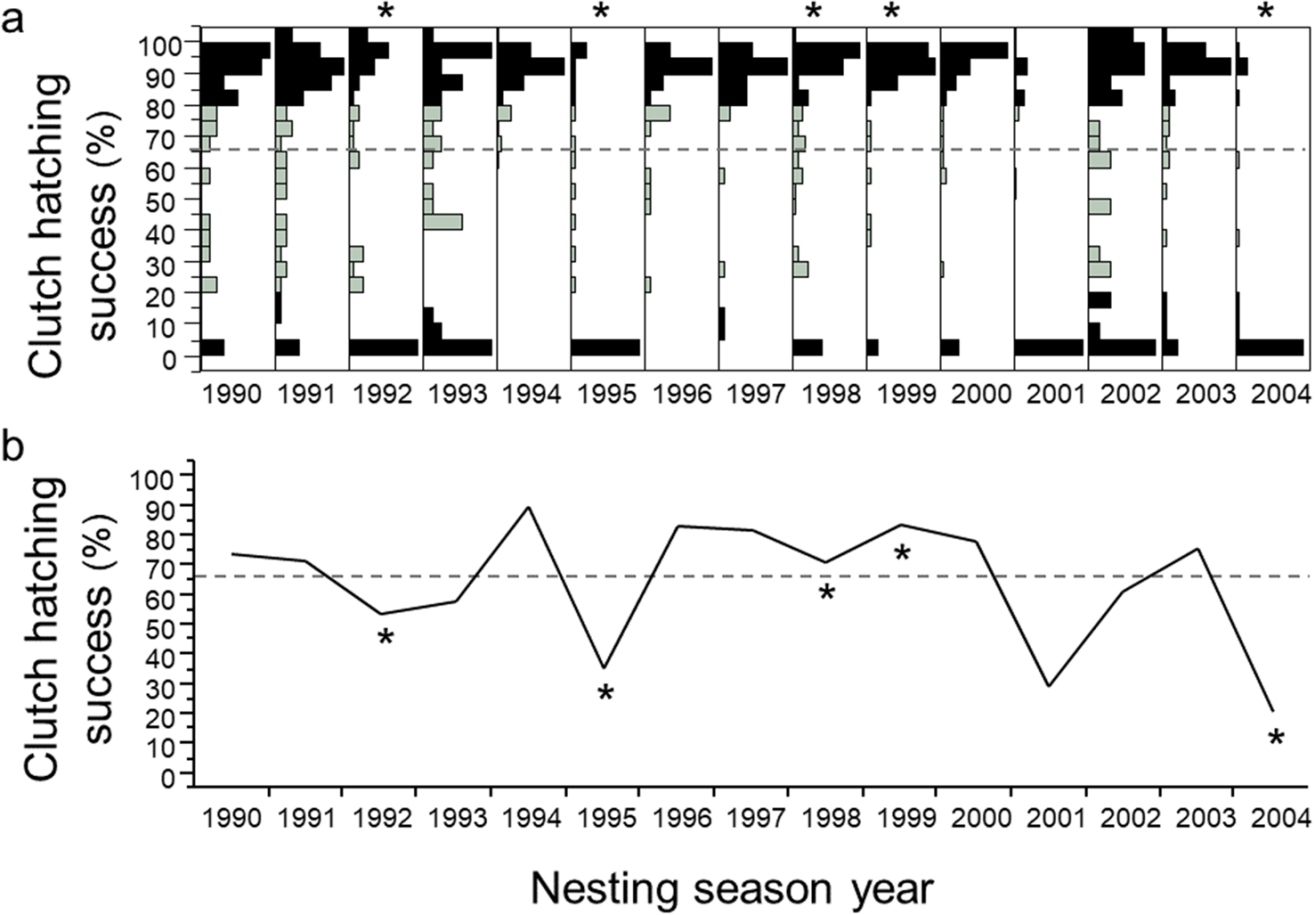
Clutch-hatching success relative to hurricane years, 1990–2004, *N* = 112 females. (**a**) the distribution of clutch-hatching success for each nesting season was bimodal. (**b**) Average clutch-hatching success for each nesting season.

**Figure 11:**
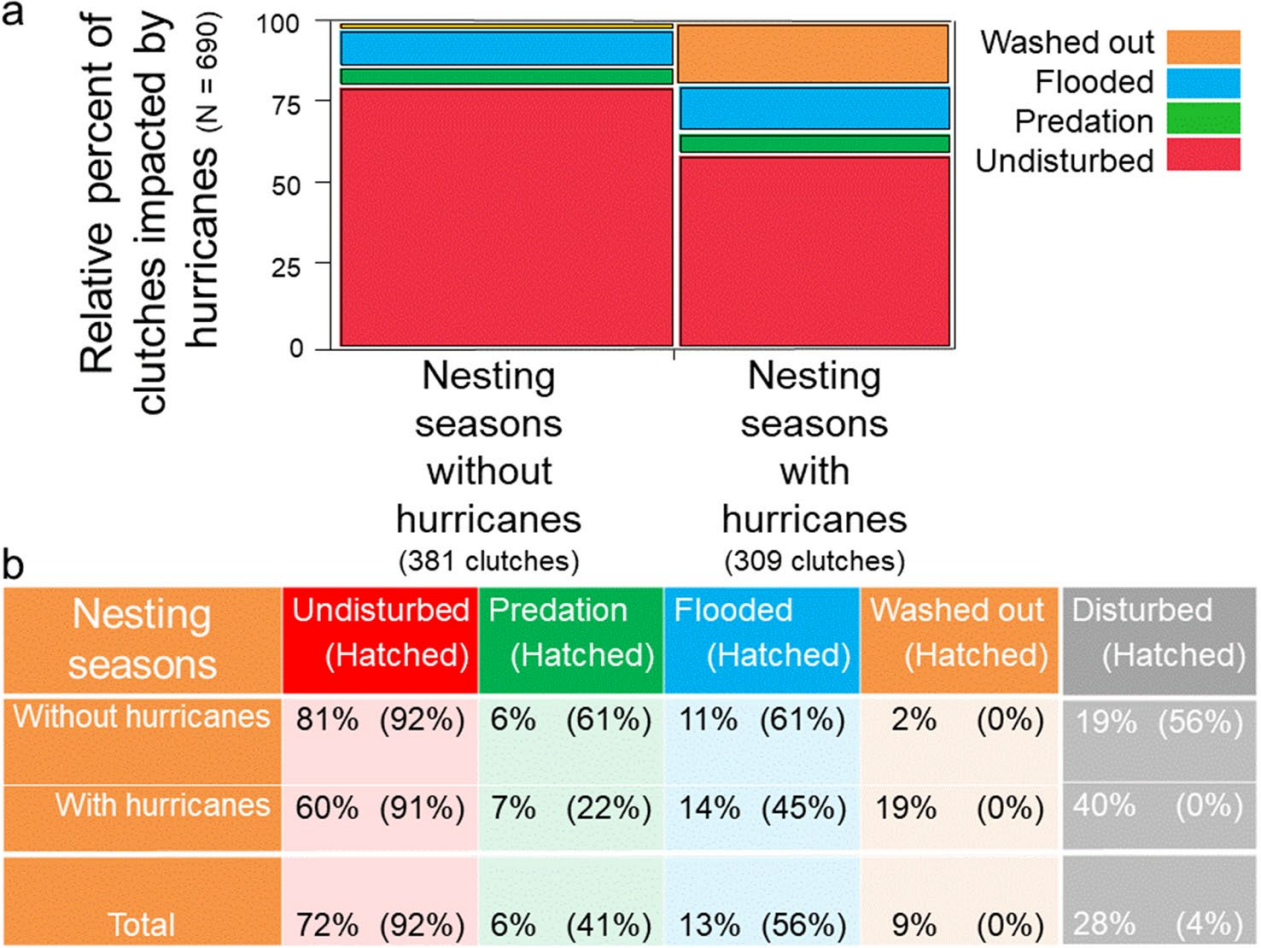
Clutch fate, 1988–2004, *N* = 112 females. (**a**) Clutch fate relative to nesting seasons with hurricanes. (**b**) Clutch fate and clutch-hatching success relative to nesting seasons with hurricanes. Percentages were rounded.

Lastly, I show that the 1995 and 2004 hurricane seasons were particularly devastating for clutches on this barrier island. High numbers of clutches were washed out, flooded or disturbed by predators (Fig. 10). Nevertheless, across five of the nesting seasons that endured ten hurricanes, 60% of clutches remained undisturbed and experienced a median hatching success exceeding 90%. For non-hurricane seasons, 81% of clutches were undisturbed, with a median hatching success exceeding 90% (Fig. 11). In the final analysis, despite hurricanes, thunderstorms and predators, the majority of clutches, 72%, experienced a median hatching success of 92%. Altogether, 66% of hatchlings survived and made it to the Gulf of Mexico. During this 17-year study, mean hatching-success for females ranged from 38 to 98%; no female experienced total reproductive failure.

## Discussion

Reproduction is a key function that differentiates biotic from abiotic systems. Here, I report a study of reproductive investments by 112 loggerhead females that nested from 1988 to 2004 on a barrier island off the Gulf coast of Naples, Florida, US. Assuming a 30-year reproductive period for mature loggerhead sea turtles, I show that nesting females remigrated to the barrier island every two-to-eight years, averaging a total of 12 nesting seasons during their reproductive lifespan^49–51^. Females produced approximately 4200 eggs, dividing them among 40 clutches. Contrary to a commonly held assumption, nesting females did not modify maternal, temporal or spatial investments in anticipation of nesting seasons with hurricanes. Moreover, only one of the eight reproductive investment strategies mitigated clutch failure—fewer clutches failed when located inside the vegetative Goldilocks zone than when located outside the zone.

Without the ability to modify clutch size, the number of clutches, the timing of clutch production or clutch location on the beach in anticipate hurricanes, how did nesting females mitigate clutch losses. I show that nesting females mitigated clutch losses by diversifying maternal, spatial and temporal investments in unpredictable patterns. As a result of confronting unpredictable risks with an overproduction of eggs and a diversified distribution of those eggs over time and space, the total number of clutches impacted by storms and predators over this 17-year study was only 28%. In the final analysis, 72% of clutches were undisturbed and experienced a median hatching success of 92%.

Although clutch-hatching success was bimodal, a small number of the disturbed clutches had an intermediate level of clutch-hatching success, averaging ~ 50%. Within the same nest, why did some eggs survive but not others? I hypothesize that eggs deposited at the top of a nest are at lower risk of mortality from flooding events than those at the bottom. Conversely, eggs deposited at the bottom of a nest are at a lower risk of mortality by predation than those at the top. Apart from nest depth and sand characteristics of nests^64–66^, little attention has been given to the role of nest morphology and the ensuing survival of some hatchlings at the expense of others. Further study is needed to confirm the role of nest morphology on intermediate levels of clutch-hatching success.

In total, I show that loggerhead females offset the loss of clutches to catastrophic storms and predators with a series of innovative reproductive adaptations. First and foremost, to meet or exceed replacement fitness, nesting females overproduced eggs. Thereafter, nesting females divided their lifetime production of eggs into clutches of diversified size, deposited clutches in diversified numbers at widely-dispersed locations on the beach at diversified intervals over multiple nesting seasons^67,68^. Although no single maternal investment strategy greatly improved clutch-hatching success, when we take into account multiple, diversified reproductive adaptations, we find that two-thirds of loggerhead sea turtle hatchlings made it to the Gulf of Mexico during this 17-year study. Moreover, no female experienced total reproductive failure during this study.

In conclusion, sea turtles have persisted through several large-scale climate changes, including ocean warming similar in magnitude to the predicted levels of ocean warming over the next 50–100 years^14–18,46,69–76^. In a cascade of consequences, as ocean warming increases, the number of severe storms will increase. Sea levels will rise. With increased storms and rise of sea levels, a larger portion of clutches will be flooded or washed out as beaches shift, erode, and disappear^77^. As a larger portion of females fail to meet replacement fitness, loggerhead sea turtle populations will decline. Without knowledge of the sea turtle’s survival and reproductive biology, we cannot develop and implement effective conservation policies. Without the implementation of well-informed conservation policies, sea turtles are likely to join the ichthyosaurs and plesiosaurs as a note in the annals of extinct marine reptiles.

## Methods

The Conservancy of Southwest Florida provided the data on loggerhead females and their clutches nesting on Keewaydin Island in the southern Gulf coast of Florida from 1988 to 2004. During the nesting and hatching season from late April through October, staff patrolled the island every night from 21:00 to 5:00 to monitor and record data on the entire nesting population of females and their clutches. After hatchlings emerged from the nest, clutch size and hatching success per clutch were determined by counting the number of empty eggshells and unhatched eggs. Hatchlings remaining in the nest were released into the Gulf. Predator type was determined by observation of injury to the eggs, the presence of tracks on the sand or, in the case of fire ants and fly larvae, the presence of the predator in the nest. After hatchlings had emerged, the remaining undeveloped eggs where examined to determine cause of death, which most often was flooding. Of the 690 nests, 61 were relocated away from the high tide line. Relocated clutches were included in the analyses of clutch size and the number of clutches, and were blocked in hatching success analyses. Data from the Conservancy were used to produce a multitude of other variables (Table 2). We did not account for the possibility that some females were interrupted in their egg production, generating falsely small clutch sizes, or the possibility that a few females produced clutches on nearby beaches, generating falsely lengthy remigration intervals or clutch intervals.

**Table 2:** Variables provided by the Conservancy of Southwest Florida and variables extrapolated for the study.

**Conservancy of Southwest Florida variables**	
1	Female identification (Tag ID number)
2	Date of clutch oviposition (day, month, year)
3	Number of eggs per clutch (clutch size)
4	Number of eggs that hatched
5	Distance of clutch from the vegetative line (converted to meters)
6	Distance of clutch from the high tide line (converted to meters)
7	Distance of female emergence along the beach (converted to meters)
8	Depth of the beach at female emergence (converted to meters)
9	Clutch fate (washed out, flooded, disturbed by a predator)
**Extrapolated variables**	
10	Clutch oviposition by month
11	Clutch oviposition by year
12	Clutch order (numeric sequence of clutches deposited per female per nesting season)
13	Number of clutches deposited per female per nesting season
14	Number of nesting seasons per female
15	Remigration interval between nesting seasons per female
16	Clutch-hatching success (percent)
17	Presence or absence of hurricanes per nesting season (yes, no)
18	Clutch fate (disturbed, undisturbed)
19	Clutch fate by predation (yes, no)
20	Clutch fate by storms (washed out, flooded)

Data were analyzed using JMP Statistical Software. Density maps were composed using JMP’s quantile-density-contours function. Because the distribution for percent hatching success was bimodal, I reported “median” hatching success rather than “mean”. For comparative analyses on skewed or bimodal data, I used non-parametric tests. Frequency distributions were displayed as histograms. Regression, ANOVA, non-parametric Wilcoxon tests, Pearson Chi Square tests, and mixed, multi-factor and multi-variable models were employed depending on data type and the distribution’s normality and variance. Figures were generated using JMP Statistical Software and PowerPoint. The hurricane map was generated from NOAA’s Historical Hurricane Tracks, a free online tool that allows users to track historic hurricane tracks (https://oceanservice.noaa.gov/news/historical-hurricanes).

### Animal welfare

Data were acquired by staff from the Conservancy of Southwest Florida program from 1988 through 2004. Staff collected data in accordance with relevant guidelines and regulations established by NOAA and the Florida Fish and Wildlife Commission. No animals were harmed during the acquisition of these data.

## Data Availability

Data from this study are available in Excel format upon request.
